# Which Non-Pharmaceutical Primary Care Interventions Reduce Inequalities in Common Mental Health Disorders? A Protocol for a Systematic Review of Quantitative and Qualitative Studies

**DOI:** 10.3390/ijerph182412978

**Published:** 2021-12-09

**Authors:** Louise Tanner, Sarah Sowden, Madeleine Still, Katie Thomson, Clare Bambra, Josephine Wildman

**Affiliations:** 1 Population Health Sciences Institute, Newcastle University, Newcastle NE1 8PB, UK; sarah.sowden@newcastle.ac.uk (S.S.); madeleine.still@newcastle.ac.uk (M.S.); katie.thomson@newcastle.ac.uk (K.T.); clare.bambra@newcastle.ac.uk (C.B.); josephine.wildman@newcastle.ac.uk (J.W.); 2 National Institute for Health Research (NIHR) Applied Research Collaboration (ARC) for the North-East and North Cumbria (NENC), Newcastle Upon Tyne NE3 3XT, UK

**Keywords:** mental disorders, healthcare disparities, primary health care, systematic review, health inequalities, PROGRESS-Plus

## Abstract

Common mental health disorders (CMDs) represent a major public health concern and are particularly prevalent in people experiencing disadvantage or marginalisation. Primary care is the first point of contact for people with CMDs. Pharmaceutical interventions, such as antidepressants, are commonly used in the treatment of CMDs; however, there is concern that these treatments are over-prescribed and ineffective for treating mental distress related to social conditions. Non-pharmaceutical primary care interventions, such as psychological therapies and “social prescribing”, provide alternatives for CMDs. Little is known, however, about which such interventions reduce social inequalities in CMD-related outcomes, and which may, unintentionally, increase them. The aim of this protocol (PROSPERO registration number CRD42021281166) is to describe how we will undertake a systematic review to assess the effects of non-pharmaceutical primary care interventions on CMD-related outcomes and social inequalities. A systematic review of quantitative, qualitative and mixed-methods primary studies will be undertaken and reported according to the PRISMA-Equity guidance. The following databases will be searched: Assia, CINAHL, Embase, Medline, PsycInfo and Scopus. Retrieved records will be screened according to pre-defined eligibility criteria and synthesised using a narrative approach, with meta-analysis if feasible. The findings of this review will guide efforts to commission more equitable mental health services.

## 1. Introduction

Common mental health disorders (CMDs), such as depressive disorders and anxiety disorders, are a major global healthcare problem, causing a large amount of suffering and imposing huge economic costs; for example, mental health problems are estimated to cost the global economy around GBP 105 billion a year [1]. In many countries, including the United States, Canada, Australia and European countries such as France and the UK, primary care is usually the first point of contact for people with mental health problems. Most patients with a mental health problem are seen only in primary care [2,3,4], and in the UK, mental ill health comprises a third of GP appointments [5]. Pharmaceutical interventions, such as antidepressants, are commonly used, and are frequently effective in the treatment of CMDs. However, there is concern amongst healthcare professionals that pharmaceutical treatments are over-prescribed or inappropriately used, resulting in the medicalising of everyday stresses and distress caused by socioeconomic deprivation [6,7,8]. Antidepressant prescriptions show an increasing trend [9] that has outpaced the rise in the prevalence of CMDs [10]. There is also evidence that some indicators of disadvantage and marginalisation, such as unemployment, are associated with increased antidepressant use independent of a diagnosis of depression [11].

Non-pharmaceutical interventions provide alternative treatment options for mental distress. In England, for example, the Improving Access to Psychological Treatment (IAPT) service was developed by the National Health Service with the aim of integrating psychological therapies into primary care [12,13]. More recently, “social prescribing” is being formally embedded into primary care to provide an alternative for patients with mental health disorders (and other chronic health conditions) [14]. Social prescribing aims to improve patients’ health and wellbeing by offering them support and linkage to community-based services that provide support with social needs and health behaviours [15,16].

With the aim of applying an equity lens to healthcare interventions, the Cochrane and Campbell Equity Methods group developed the PROGRESS-Plus framework to identify a range of sociodemographic characteristics that stratify health outcomes [17]. A number of PROGRESS-Plus domains have been found to be associated with the prevalence of mental health problems. There are sex differences in rates of mental ill health, with women having higher rates of anxiety and depression [18] and higher rates of substance-abuse-related mental health problems in men [19]. Mental health outcomes have also been found to be associated with one’s place of residence, including in terms of access to green space [20] and living in areas of socioeconomic disadvantage [21]. Differential rates of mental health problems have also been found to be associated with race and ethnicity [22,23,24], occupation [25,26,27], religious identity [28,29,30], social capital [31], educational attainment [27,30], age [30], disability status [32] and sexual orientation [33]. Mental ill health is a particular problem in areas of socioeconomic deprivation, where mental health problems can be both a cause and effect of poverty and of social problems such as unemployment, homelessness, debt and violence [30].

In addition to experiencing higher rates of CMDs, people living with disadvantage and marginalisation are less able to access and benefit from treatments for conditions such as anxiety and depression [16,34]. In the UK, as in other high-income countries [35], addressing both mental ill health and health inequalities are key policy objectives, as evidenced in the NHS Long-term Plan [36] and the narrative surrounding the NHS response to the COVID-19 pandemic [37]. For these reasons, there is a pressing need for evidence about which interventions will reduce inequalities in treatment outcomes and which may, unintentionally, increase them. This systematic review will examine evidence on primary care interventions that are likely to decrease, or potentially increase, health inequalities in treatment access and outcomes for patients experiencing CMDs. Findings will guide the commissioning of more equitable mental health services.

**Figure 1:**
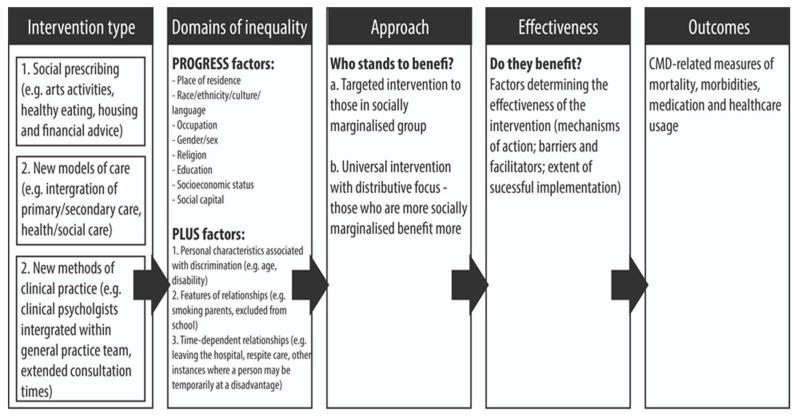
Framework for addressing inequalities in CMD-related health outcomes in relation to the PROGRESS-Plus domains [17,43,44], adapted from Sowden et al. [41].

In line with PRISMA-E guidelines [38,39,40], as the first stage of this equity-focused review, a framework for conceptualising primary care interventions and mental health inequalities was developed (Figure 1). The framework outlines the three types of non-pharmaceutical interventions considered in this review:

1. Social prescribing (for example, arts activities, healthy eating, housing and financial advice);

2. New models of care (for example, integration of primary and secondary healthcare, or the integration of health and social care);

3. New methods of clinical practice (for example, clinical psychologists integrated with general practice teams or extended consultation times).

The framework considers the potential domains of inequality addressed by an intervention, the approach taken, and factors that may impact the effectiveness of any given intervention in reducing inequalities in mental health outcomes. It was developed based on an existing framework used in a previous equity-focused review [41] and will be revised iteratively [42] as evidence from the systematic review emerges.

## 2. Materials and Methods

The review will be carried out following established criteria for the good conduct and reporting of equity-focused systematic reviews using PRISMA-E guidelines [38,39,40] and reporting here conforms to the standards of the Preferred Reporting Items for Systematic Reviews and Meta-analysis Protocols (PRISMA-P) (see Supplementary File S1) [45]. The protocol for this systematic review was registered on the PROSPERO database on 23rd September 2021 (registration number: CRD42021281166) [46].

### 2.1. Research Questions

The main research question to be addressed in this review is:

Which non-pharmaceutical primary care interventions reduce inequalities in CMD-related adverse health outcomes?

Review sub-questions are:

1. Which non-pharmaceutical primary care interventions reduce the occurrence of CMD-related adverse health outcomes amongst populations in the most disadvantaged groups in relation to the PROGRESS-Plus framework?
2. Which non-pharmaceutical primary care interventions reduce inequalities in CMD- related adverse health outcomes between people from the least and most disadvantaged groups?
3. What are the mechanisms by which non-pharmaceutical primary care interventions impact CMD-related adverse health outcomes and inequalities?
4. What are the barriers and facilitators to the implementation of non-pharmaceutical primary care interventions in disadvantaged groups?

### 2.2. Objectives

The objectives of this systematic review are to:

- Locate studies reporting data for the effects of non-pharmaceutical primary care interventions on CMD-related health outcomes and inequalities, using systematic searches of bibliographic databases and sources of grey literature and systematic screening methods;
- Quantify the effects of non-pharmaceutical primary care interventions on CMD-related health outcomes and inequalities;
- Identify which aspects of the identified interventions influence CMD-related health outcomes and inequalities and the mechanisms (including barriers and facilitators) by which these factors exert their effects;
- Assess the methodological quality of the synthesised evidence;
- Identify policy implications and areas for further research in relation to the review findings.

### 2.3. Inclusion Criteria

The following criteria (summarised in Table 1) will be applied to each full text in order to assess their eligibility for inclusion in the review.

#### 2.3.1. Population

The population of interest consists of people who are being treated in primary care in any high-income country defined by The Organisation for Economic Co-operation and Development (OECD) [47] whose characteristics in relation to one or more of the PROGRESS-Plus factors [48] are reported. Included studies must indicate that all or some participants have a CMD, which must be one of the following disorders, defined by Lund (2020): anxiety, depression, somatoform disorder, post-traumatic stress disorder or postnatal depression [49]. Participants are not required to have received a CMD diagnosis; presence of a CMD may be indicated using data from mental health screening tools (e.g., the Self-Reporting Questionnaire (SRQ-20)). The mental health status of participants may be reported narratively (e.g., in the title, participant characteristics or inclusion criteria) or in the baseline characteristics table. We will exclude studies exclusively involving participants with the following more severe and less common conditions, defined by Lund (2020): psychosis, dementias, child and adolescent mental disorders, conversion disorders, body dysmorphic disorders, personality disorders, eating disorders, suicide, self-harm, substance use disorders, intellectual disability, epilepsy and developmental disorders [49].

We will include studies reporting interventions that have been delivered exclusively to a disadvantaged population subgroup covered by the PROGRESS-Plus criteria (e.g., where participants are all from an ethnic minority group). We will also include studies involving participants with other specific characteristics (e.g., persons with specific exposures such as victims of abuse) if one or more of their PROGRESS-Plus characteristics are also reported. Furthermore, studies that report interventions that have been delivered universally to people from disadvantaged and non-disadvantaged backgrounds (e.g., older versus younger persons; individuals from low- versus high-income households) will be included if the authors report a sub-group analysis of the differential effectiveness of the intervention between population sub-groups (e.g., persons with and without disabilities).

#### 2.3.2. Intervention

Interventions delivered by or referred to from primary care teams (including GPs and allied health professionals based in GP practices and community pharmacies) will be included.

For the purpose of this review, a broad definition of non-pharmaceutical primary care interventions will be used, including referral of individuals to activities and support services provided by the voluntary sector as well as new models of care or methods of clinical practice in relation to patient care. We will include psychological interventions, such as Cognitive Behavioural Therapy.

Studies exclusively investigating the effects of pharmaceutical interventions will be excluded. However, where a pharmaceutical intervention is one component of a multifaceted, integrative, holistic approach to treatment and care, the overall intervention will be included.

The intervention must have been either delivered exclusively to a disadvantaged population sub-group covered by the PROGRESS-PLUS criteria (e.g., older persons; individuals with disabilities) or reported a sub-group analysis of the differential effectiveness of the intervention between population sub-groups (e.g., older versus younger adults; persons with and without disabilities).

#### 2.3.3. Comparators

##### Population

We will include studies reporting data on CMD-related health outcomes in relation to at least one type of inequality from the PROGRESS-PLUS criteria [48], i.e., place of residence (e.g., rural/urban location); race/ethnicity/culture/language; occupation; gender/sex; religion; education; socioeconomic status including social capital [48].

Eligible studies may present data enabling between-group comparisons (e.g., between persons who received the intervention from low- versus high-income households) or within-group comparisons (e.g., before and after the intervention, amongst individuals who are all from low-income households).

##### Intervention

Data from included studies comparing the effectiveness of intervention versus no or alternative intervention (including alternative similar interventions and variations in the format, duration and intensity of the intervention as well as usual treatment vs. novel one) will also be extracted where available and included in the synthesis.

#### 2.3.4. Outcomes

Relevant outcomes from quantitative studies will include measures of morbidity which are directly related to CMDs (e.g., the number of health care consultations and measures of medication usage for CMDs) in addition to assessments from validated mental health screening tools, including but not limited to the State–Trait Anxiety Inventory (STAI) [50], the Perceived Stress Scale (PSS) [51], the Positive and Negative Affect Schedule (PANAS) [52], the Warwick–Edinburgh Mental Well-being Scale (WEMWBS) [53], the Self-Reporting Questionnaire (SRQ-20) [54] or the General Health Questionnaire (GHQ) [55]. We will also include adapted versions of these mental health screening tools that have been validated in other languages (e.g., the Spanish and Dutch versions of the PANAS) [56,57], as well as validated tools that have been developed for use in high-income countries outside of the UK, if the study is reported in English.

The overall impact of the interventions of interest will be assessed by comparing the occurrence of CMD-related adverse health outcomes before and after the intervention amongst health-disadvantaged population subgroups. Health inequalities will be assessed by comparing CMD-related adverse health outcomes between the most and least health-disadvantaged groups.

From qualitative studies, we will extract information providing insights on the mechanisms by which non-pharmaceutical primary care interventions could impact CMD outcomes and inequalities, in addition to barriers and facilitators to the successful implementation of these programmes.

#### 2.3.5. Study Design

Quantitative primary studies, including randomised controlled trials (RCTs), other intervention studies (e.g., quasi-experimental), longitudinal studies (e.g., cohort and panel studies), repeated cross-sectional studies and ecological studies [58], will be included in the review in addition to qualitative [59] and mixed-methods primary studies.

#### 2.3.6. Context

In order to be included in the review, studies must be written in English language and have been published in an OECD high-income county (studies that do not meet these criteria will be excluded during screening) [47].

### 2.4. Search Strategy

The following databases will be searched from their start until 1 June 2021 (host platforms in brackets): Applied Social Sciences Index and Abstracts (ASSIA; ProQuest)(Ann Arbor MI, USA); Cumulative Index to Nursing and Allied Health Literature (CINAHL) (EBSCO, USA); Embase (Ovid, London, UK); PsycInfo (EbscoHost, Ipswich MA, USA); Scopus (Elsevier, Amsterdam, The Netherlands). The draft search strategy for Medline is shown in Supplementary File S2.

The useful resource list of the Social Prescribing Network [60], the Social Interventions Research and Evaluations Network (SIREN) [61] and relevant charity websites will also be purposefully searched for relevant articles. Citing references will also be identified using Google Scholar’s “cited by” feature. We will also screen the reference lists of reviews located during the searches which are deemed relevant to the research question, as well as any primary studies which are included in the review, to identify further potentially relevant studies. No limits on date or language will be placed on the searches.

Search strings for the relevant databases were built from existing search filters for PROGRESS-Plus [43] and mental health components [62]. Primary health care elements of the search were taken from that used in a Cochrane review of primary care treatment for alcohol and drugs [63]. The search strings for each database will be peer-reviewed by an experienced information specialist, using the PRESS checklist [64] prior to implementation.

### 2.5. Screening and Selection

Records located in the searches will be downloaded into an Endnote [65] library and de-duplicated. Rayyan software (Qatar Computing Research Institute, HBKU, Doha, Qatar)will be used to screen studies retrieved from the literature searches [66]. A two-stage process will be used to identify studies for inclusion in the review. First, titles and abstracts will be screened to identify studies relevant to the review topic. The full texts of potentially relevant studies will be sourced and assessed for eligibility in relation to pre-defined inclusion and exclusion criteria. One reviewer (MS) will screen each record, and a second member of the review team (LT) will check a random 10% sample at both stages of the screening process. Screening conflicts will be resolved via discussion and adjudication by a third reviewer (JMW, KT or SS) where necessary.

### 2.6. Data Extraction

Separate data-extraction forms will be created for quantitative, qualitative and mixed-methods studies. These will be based on existing tools and pre-piloted on a sample of studies deemed eligible for inclusion in the review, with modifications to ensure that all relevant information is captured. Extracted information from quantitative studies will include citation details (first author name and publication date), study characteristics (study aims, design, country and setting), population characteristics (PROGRESS-Plus and other reported characteristics), intervention details (type of intervention, mode and duration of delivery), comparators (pertaining to the population and intervention), outcomes and results (mean and SD values for each comparison group continuous data; number of events and sample size for each comparison group for categorical outcomes). We will extract additional data reported in the included studies which quantify the association between the exposures and outcomes of interest (e.g., results from correlational, regression and modelling studies). The qualitative-data-extraction form will include bibliographic information, methods (e.g., number of participants, data collection method), relevant findings, illustrations from the paper (e.g., participant quotes) and a suggested category or code for that finding. Data from each study will be extracted by one person and checked by a second person.

### 2.7. Quality Appraisal

Appropriate tools will be selected based on the study designs identified for inclusion in the review. The CASP tools [67] will be used to assess the quality of quantitative studies; a modified version of the relevant CASP tool will be used to quality appraise qualitative studies [68]. If eligible mixed-methods studies are identified, these will be critically appraised using the Mixed Methods Appraisal Tool (MMAT) [69]. Repeated cross-sectional studies will be assessed using the Appraisal tool for Cross-Sectional Studies (AXIS) [70]. All studies will be synthesised regardless of quality. A nominal scoring system will be devised to enable comparability of the overall quality between studies collecting the same type of data (i.e., quantitative, qualitative and mixed-methods). Based on the scoring system developed by the Cochrane Collaboration, studies will be rated as low quality, some concerns or high quality based on domain scores [71].

## 3. Results

### Synthesis

The review will be reported according to the PRISMA-Equity guidance [40]. We will include a paragraph summarising the overall characteristics of included studies, including the number and percentage of studies with different characteristics (e.g., types of study, country of publication and participant characteristics). It is anticipated that heterogeneity will prevent the implementation of meta-analysis. However, if the quantitative data allow, pairwise meta-analysis will be performed using RevMan 5 [72]. For continuous outcomes, we will compare mean values, whereas the number of events and sample size will be extracted for binary outcomes to determine outcome rates. For studies presenting within-group comparisons (where an intervention has been delivered exclusively to a disadvantaged subgroup), we will compare mean values for continuous outcomes (e.g., mean anxiety scores) before versus after the intervention. For studies presenting between-group comparisons, we will compare changes from baseline values (where the data are available) for continuous variables and post-intervention data for binary variables (e.g., GP consultation rates) between population sub-groups. For the between-group comparisons, where there are data for >2 categories for a particular domain of inequality (e.g., low-, middle- and high-income households), we will compare the most and least disadvantaged population subgroups (e.g., highest- versus lowest-income households). Statistical heterogeneity will be assessed using the Chi^2^ and Higgins I^2^ statistics. Heterogeneity will be deemed to be present if the *p* value for the Chi^2^ test is <0.10 or the Higgins I^2^ statistic is >50%. A random-effects model will be implemented. If it is not feasible to undertake meta-analysis of the quantitative data, a vote-counting approach, such as a Harvest plot, will be used to graphically present the quantitative data. [73]

Thomas and Harden’s (2008) three-stage approach to qualitative synthesis [74] will be used to thematically synthesise qualitative data. This will involve (1) coding the data line by line according to content and meaning; (2) grouping codes according to similarities and differences to produce descriptive themes; (3) generating analytical themes according to the reviewer’s interpretation of the data in relation to the review question.

The results will be presented in relation to each domain of inequality for which relevant data were identified. The domain of inequality we are primarily interested in in this systematic review is socioeconomic status. Data in relation to this characteristic will be included in the synthesis preferentially over other PROGRESS-plus factors, if including evidence in relation to other social factors would be unmanageable within the time constraints of this project.

The Economic and Social Research Council’s framework [75] will be used to write the narrative synthesis, combining the quantitative (effectiveness) results with the qualitative (mechanisms of action; barriers and facilitators) themes. This includes the following components:

- Developing a theory of how non-pharmaceutical primary care interventions reduce CMD-related adverse health outcomes and inequalities, why and for whom;
- Developing a preliminary synthesis of the data;
- Exploring relationships within and between studies;
- Assessing the robustness of the synthesis.

A Best Available Evidence approach [76] will be used to synthesise evidence from the quantitative studies. This will involve reporting evidence from studies with the most robust designs assessing the clinical effectiveness of interventions, prior to evidence from less-robust study designs. The following hierarchy will be used: (1) RCTs, other intervention studies (e.g., quasi-experimental); (2) individual-level longitudinal studies; (3) repeated cross-sectional studies; (4) ecological studies.

## 4. Discussion

CMDs are prevalent globally. In England, for example, 1 in 6 people report experiencing a common mental health problem (such as anxiety and depression) in any given week (data from 2014) [77]. Non-pharmaceutical primary care interventions provide alternative therapeutic options for mental distress to drug treatments. In order to support the use of such treatments for people with CMDs, their effectiveness must be assessed, as well as their impacts on health inequalities.

This systematic review will provide evidence regarding the effectiveness of non-pharmaceutical primary care interventions at reducing inequalities in CMD-related outcomes in relation to the PROGRESS-PLUS domains [48]. The mechanisms by which these interventions impact the outcomes of interest and barriers and facilitators to implementation will be explored. This will enable policy makers to identify non-pharmaceutical primary care interventions that are most effective at reducing inequalities in mental health outcomes and to determine which aspects of these interventions increase or decrease their effectiveness in different populations.

Limitations of the methods include potential language bias from excluding studies not published in the English language. Additionally, we will deviate from gold-standard methods by only having one reviewer screen, extract data and quality-appraise all of the articles and a second reviewer perform a 10% check.

Dissemination of findings will take place through a written report for stakeholders. The report findings will also be shared in two half-day workshops: one with practitioner stakeholders, including from the Clinical Commissioning Groups, Primary Care Networks and the Integrated Care Systems, and the other with members of the public. Dissemination workshops will also seek input from practitioners and public stakeholders into a bid for further funding. Two articles will be published in peer-reviewed journals.

## 5. Conclusions

Globally, CMDs create significant health and economic burdens. In many countries, patients with CMDs are predominantly treated in primary care. While many CMDs are treated with pharmaceutical interventions, non-pharmaceutical interventions, such as psychological therapies, are available as alternative treatments for people with anxiety and depressive disorders. Within primary care, new models of care and new methods of clinical practice, such as social prescribing, are also being developed to provide non-pharmaceutical options for patients with CMDs. However, the effects of these interventions on social inequalities in CMD-related health outcomes is unknown. This review will assess the impacts of non-pharmaceutical primary care interventions on social inequalities in CMD-related health outcomes, based on evidence from quantitative, qualitative and mixed-methods studies. The results will provide evidence to support the delivery of more equitable mental health services.

## Figures and Tables

**Table 1:** Summary of inclusion and exclusion criteria.

Inclusion	Exclusion
**Population:** People with a common mental disorder (CMD) ^a^, who are being treated in primary care.	**Population:** Studies exclusively involving participants with alternative mental or physical health conditions.
**Intervention:** Non-pharmaceutical interventions delivered by or referred to from primary care teams. These will include: Activities and support services provided by the voluntary sector; New models of care or methods of clinical practice in relation to patient care; Psychological interventions.	**Intervention:** Studies exclusively investigating the effects of pharmaceutical interventions.
**Comparators:** **Population** Comparisons before and after the intervention amongst individuals from health disadvantaged population sub-groups; Comparisons between health-disadvantaged and non-disadvantaged population sub-groups. **Intervention** Comparisons between non-pharmaceutical primary care intervention versus no or alternative intervention.	
**Outcomes:** Quantitative measures of: Healthcare use related to CMDs; Medication use related to CMDs; CMD screening and assessment tools Self-reported qualitative data on: ₋ Mechanisms by which interventions impact on mental health; ₋ Barriers and facilitators to implementing interventions.	
**Study design:** Quantitative, qualitative and mixed methods primary studies.	**Study design:** Editorials and letters.
**Context:** Studies published in English language in an OECD high-income country.	

^a^ CMDs of interest in this review are: anxiety, depression, somatoform disorders, post-traumatic stress disorder or post-natal depression.

## Supplementary Materials

The following are available online at https://www.mdpi.com/article/10.3390/ijerph182412978/s1, file S1: PRISMA-P 2015 checklist, file S2: draft search strategy for Medline.
