# Supplementary material for: Which Non-Pharmaceutical Primary Care Interventions Reduce Inequalities in Common Mental Health Disorders? A Protocol for a Systematic Review of Quantitative and Qualitative Studies
Source: Int J Environ Res Public Health. 2021 Dec 9;18(24):12978. doi: 10.3390/ijerph182412978 (PMC8701146; doi:10.3390/ijerph182412978)
Supplement: Supplementary file 1 [file ijerph-18-12978-s001.zip › Supplementary file S1 PRISMA-P 2015 checklist.pdf]

PRISMA-P 2015 Checklist

This checklist has been adapted for use with protocol submissions to *Systematic Reviews* from Table 3 in Moher D et al: Preferred reporting items for systematic review and meta-analysis protocols (PRISMA-P) 2015 statement. *Systematic Reviews* 2015 **4**:1

Section/topic	#	Checklist item	Information reported		Page number(s)
			Yes	No	
**ADMINISTRATIVE INFORMATION**					
**Title**					
Identification	1a	Identify the report as a protocol of a systematic review	x		1
Update	1b	If the protocol is for an update of a previous systematic review, identify as such	NA		
**Registration**	2	If registered, provide the name of the registry (e.g., PROSPERO) and registration number in the Abstract	x		1
**Authors**					
Contact	3a	Provide name, institutional affiliation, and e-mail address of all protocol authors; provide physical mailing address of corresponding author	x		1
Contributions	3b	Describe contributions of protocol authors and identify the guarantor of the review	x		11
**Amendments**	4	If the protocol represents an amendment of a previously completed or published protocol, identify as such and list changes; otherwise, state plan for documenting important protocol amendments	NA		
**Support**					
Sources	5a	Indicate sources of financial or other support for the review	x		10-11
Sponsor	5b	Provide name for the review funder and/or sponsor	x		10-11
Role of sponsor/funder	5c	Describe roles of funder(s), sponsor(s), and/or institution(s), if any, in developing the protocol	x		11
**INTRODUCTION**					
**Rationale**	6	Describe the rationale for the review in the context of what is already known	x		1-3
**Objectives**	7	Provide an explicit statement of the question(s) the review will address with reference to participants, interventions, comparators, and outcomes (PICO)	x		3-4

BioMed Central
The Open Access Publisher

Section/topic	#	Checklist item	Information reported		Page number(s)
			Yes	No	
**METHODS**					
**Eligibility criteria**	8	Specify the study characteristics (e.g., PICO, study design, setting, time frame) and report characteristics (e.g., years considered, language, publication status) to be used as criteria for eligibility for the review	x		5-8
**Information sources**	9	Describe all intended information sources (e.g., electronic databases, contact with study authors, trial registers, or other grey literature sources) with planned dates of coverage	x		4
**Search strategy**	10	Present draft of search strategy to be used for at least one electronic database, including planned limits, such that it could be repeated	x		Supplementary file S2
STUDY RECORDS					
Data management	11a	Describe the mechanism(s) that will be used to manage records and data throughout the review	x		4-5
Selection process	11b	State the process that will be used for selecting studies (e.g., two independent reviewers) through each phase of the review (i.e., screening, eligibility, and inclusion in meta-analysis)	x		4-5
Data collection process	11c	Describe planned method of extracting data from reports (e.g., piloting forms, done independently, in duplicate), any processes for obtaining and confirming data from investigators	x		8
**Data items**	12	List and define all variables for which data will be sought (e.g., PICO items, funding sources), any pre-planned data assumptions and simplifications	x		8
**Outcomes and prioritization**	13	List and define all outcomes for which data will be sought, including prioritization of main and additional outcomes, with rationale	x		8
**Risk of bias in individual studies**	14	Describe anticipated methods for assessing risk of bias of individual studies, including whether this will be done at the outcome or study level, or both; state how this information will be used in data synthesis	x		8-9
DATA					
**Synthesis**	15a	Describe criteria under which study data will be quantitatively synthesized	x		9-10
	15b	If data are appropriate for quantitative synthesis, describe planned summary measures, methods of handling data, and methods of combining data from studies, including any planned exploration of consistency (e.g., I^2, Kendall's tau)	x		9
	15c	Describe any proposed additional analyses (e.g., sensitivity or subgroup analyses, meta-regression)	x		9
	15d	If quantitative synthesis is not appropriate, describe the type of summary planned	x		9

Section/topic	#	Checklist item	Information reported		Page number(s)
			Yes	No	
**Meta-bias(es)**	16	Specify any planned assessment of meta-bias(es) (e.g., publication bias across studies, selective reporting within studies)		x	
**Confidence in cumulative evidence**	17	Describe how the strength of the body of evidence will be assessed (e.g., GRADE)		x	

BioMed Central
The Open Access Publisher
