# Supplementary material for: Which Non-Pharmaceutical Primary Care Interventions Reduce Inequalities in Common Mental Health Disorders? A Protocol for a Systematic Review of Quantitative and Qualitative Studies
Source: Int J Environ Res Public Health. 2021 Dec 9;18(24):12978. doi: 10.3390/ijerph182412978 (PMC8701146; doi:10.3390/ijerph182412978)
Supplement: Supplementary file 1 [file ijerph-18-12978-s001.zip › Supplementary file S2.pdf]

Ovid MEDLINE(R) and In-Process, In-Data-Review & Other Non-Indexed Citations <1946 to September 10, 2021>

1 Residence Characteristics/

2 Environment design/

3 exp Marital status/

4 neighbo?rhood*.mp.

5 residential environment*.mp.

6 rural*.mp.

7 inner?city.mp.

8 housing instability.mp.

9 housing insecurity.mp.

10 housing strain.mp.

11 housing security.mp.

12 mortgage problems.mp.

13 foreclosure.mp.

14 eviction*.mp.

15 housing loss.mp.

16 home repossession.mp.

17 home ownership.mp.

18 (repossess* adj3 hous*).mp.

19 (repossess* adj3 propert*).mp.

20 mortgage delinquency.mp.

21 mortgage arrears.mp.

22 mortgage debt*.mp.

23 overcrowding.mp.

24 (living adj1 (outside or inside or near* or adjacent)).mp.

25 (household adj2 size).mp.

26 (marital status or marriage status).mp.

27 (widow* or cohabit* or divorce* or single parent* or live* alone).mp.

28 or/1-27

29 Cultural Deprivation/

30 Acculturation/

31 Culture/

32 Cultural Characteristics/ or Cross-Cultural Comparison/

33 Cultural Diversity/

34 Language/

35 "Transients and Migrants"/

36 exp "Emigrants and Immigrants"/

37 Minority groups/

38 Minority health/

39 Prejudice/

40 Racism/

41 Xenophobia/

42 Social Discrimination/

43 exp Race Relations/

44 exp Ethnic Groups/

45 exp Continental Population Groups/

46 Refugees/

47 minorit*.mp.

48 migration background.mp.

49 racial.mp.

50 racism.mp.

51 ethnology.mp.

52 race.mp.

53 ethnic*.mp.

54 non?English.mp.

55 language other than.mp.

56 latino*.mp.

57 latina.mp.

58 hispanic*.mp.

59 whites.mp.

60 blacks.mp.

61 Black*.mp.

62 caucasian*.mp.

63 non?white.mp.

64 Torres Strait Islander.mp.

65 aboriginal.mp.

66 native american.mp.

67 inuit.mp.

68 eskimo.mp.

69 first nation*.mp.

70 indigenous.mp.

71 english as a second language.mp.

72 foreign language.mp.

73 gypsy.mp.

74 traveller.mp.

75 or/29-74

76 Occupations/

77 Unemployment/

78 occupations.mp.

79 unemployment.mp.

80 or/76-79

81 exp Gender Identity/

82 Women's Health/

83 gender differences.mp.

84 (sex disparit* or sex difference?).mp.

85 gender role.mp.

86 sex role.mp.

87 wom#n* role?.mp.

88 m#n* role?.mp.

89 servicewomen.mp.

90 transgender.mp.

91 Sex factors/

92 or/81-91

93 exp Educational status/

94 Education/

95 Schooling.mp.

96 educational status.mp.

97 (education* adj2 level?).mp.

98 ((higher or better or worse or less) adj educated).mp.

99 ((higher or better or worse or less) adj level? of education).mp.

100 or/93-99

101 Religion/

102 religi*.mp.

103 or/101-102

104 Social determinants of Health/

105 Psychosocial Deprivation/

106 Sociological Factors/

107 Working Poor/

108 Hierarchy, Social/

109 disparit*.mp.

110 inequalit*.mp.

111 inequit*.mp.

112 equity.mp.

113 deprivation.mp.

114 gini.mp.

115 concentration index.mp.

116 Socioeconomic Factors/

117 Social Welfare/

118 exp Social Class/

119 exp Poverty/

120 Income/

121 Social class*.mp.

122 social determinants.mp.

123 social status.mp.

124 social position.mp.

125 social background.mp.

126 social circumstance.mp.

127 socio-economic.mp.

128 socioeconomic.mp.

129 sociodemographic.mp.

130 socio-demographic.mp.

131 SES.mp.

132 disadvantaged.mp.

133 impoverished.mp.

134 poverty.mp.

135 economic level.mp.

136 assets index.mp.

137 income*.mp.

138 or/104-137

139 Social Stigma/

140 social capital/

141 Social Control, Informal/

142 exp Social Support/

143 exp Social Environment/

144 Trust/

145 Social conditions/

146 Social isolation/

147 Social marginalization/

148 Anomie/

149 social participation/

150 social exclusion.mp.

151 (social adj (capital or cohes* or organis* or organiz*)).mp.

152 (community adj3 (cohes* or participa*)).mp.

153 ((neighbourhood or neighborhood) adj cohes*).mp.

154 social relationships.mp.

155 social network*.mp.

156 collective efficacy.mp.

157 civil society.mp.

158 informal social control.mp.

159 neighbo*rhood disorder.mp.

160 social disorgani?ation.mp.

161 anomie.mp.

162 social support.mp.

163 social participation.mp.

164 trust.mp.

165 emotional support.mp.

166 psychosocial support.mp.

167 community capital.mp.

168 neighbo*rhood cohesion.mp.

169 social influence.mp.

170 (soci*context* or soci*-context*).mp.

171 or/139-170

172 Health Status Disparities/

173 Health Services Accessibility/

174 Health Equity/

175 health*care disparit*.mp.

176 health care disparit*.mp.

177 health status disparit*.mp.

178 health disparit*.mp.

179 health inequalit*.mp.

180 (health inequit* or medically underserved).mp.

181 or/172-180

182 28 or 75 or 80 or 92 or 100 or 103 or 138 or 171 or 181

183 potential determinants.mp.

184 significant correlates of.mp.

185 (independent correlates or independent association*).mp.

186 variables associated with.mp.

187 determinants of.mp.

188 factors associated with.mp.

189 identif* determinants.mp.

190 (more likely or less likely or just as likely).mp.

191 risk factors for.mp.

192 (significantly related to or significant predictor).mp.

193 (also adj2 associated with).mp.

194 (at increased risk or at decreased risk).mp.

195 association* between.mp.

196 (positively associated or negatively associated).mp.

197 differed by.mp.

198 (were high* amongst or were low* amongst).mp.

199 (inverse relationship with or inversely associated with or inversely related to).mp.

200 reverse association.mp.

201 differentially affects.mp.

202 evidence of a link between.mp.

203 (significantly adj3 likelihood of).mp.

204 protective factors for.mp.

205 (differ* adj2 according to).mp.

206 (positive adj2 gradient).mp.

207 ((negative adj2 gradient) or (inverse adj2 gradient)).mp.

208 (trends were adj3 across).mp.

209 (related to adj3 variable*).mp.

210 (differences were adj3 explained by).mp.

211 (significant among or no# significant among).mp.

212 or/182-211

213 181 or 212

214 depress$.mp.

215 adolescent$.mp.

216 exp Mental disorders/

217 psych$.mp.

218 "use disorder".tw.

219 behav$.mp.

220 exp psychotropic drugs/

221 exp psychology, social/

222 neuro$.mp.

223 dt.fs.

224 exp brain diseases/

225 cognitive$.mp.

226 exp neurotransmitter agents/

227 exp psychotherapy/

228 exp social problems/

229 anxiety.mp.

230 exp anxiety/

231 attention.mp.

232 exp emotions/

233 exp neurobehavioral manifestations/

234 chronic.tw.

235 mental health.mp.

236 stress.mp.

237 exp stress, psychological/

238 alcohol.mp.

239 abus$.mp.

240 prevent.mp.

241 exp adaptation, psychological/

242 outcome measure.tw.

243 exp mental health services/

244 or/214-243

245 213 and 244

246 exp General Practice/

247 Primary Health Care/
248 Community Health Services/
249 exp Emergency Service, Hospital/
250 Physicians, Primary/ or Physicians, Family/
251 General Practitioners/
252 (general pract$ or family pract$ or GP or physician$ or emergency department$ or emergency room$ or trauma care or "accident and emergency" or community).tw.
253 ((family or community or practice$) adj (medic$ or doctor$ or physician$ or health$ or nurs$)).tw.
254 (primary adj2 care).tw.
255 shared care.tw.
256 or/246-255
257 245 and 256
258 (afghanistan or albania or algeria or american samoa or angola or "antigua and barbuda" or antigua or barbuda or argentina or armenia or armenian or aruba or azerbaijan or bahrain or bangladesh or barbados or republic of belarus or belarus or byelarus or belorussia or byelorussian or belize or british honduras or benin or dahomey or bhutan or bolivia or "bosnia and herzegovina" or bosnia or herzegovina or botswana or bechuanaland or brazil or brasil or bulgaria or burkina faso or burkina fasso or upper volta or burundi or urundi or cabo verde or cape verde or cambodia or kampuchea or khmer republic or cameroon or cameron or cameroun or central african republic or ubangi shari or chad or chile or china or colombia or comoros or comoro islands or iles comores or mayotte or democratic republic of the congo or democratic republic congo or congo or zaire or costa rica or "cote d'ivoire" or "cote d' ivoire" or cote divoire or cote d ivoire or ivory coast or croatia or cuba or cyprus or czech republic or czechoslovakia or djibouti or french somaliland or dominica or dominican republic or ecuador or egypt or united arab republic or el salvador or equatorial guinea or spanish guinea or eritrea or estonia or eswatini or swaziland or ethiopia or fiji or gabon or gabonese republic or gambia or "georgia (republic)" or georgian or ghana or gold coast or gibraltar or greece or grenada or guam or guatemala or guinea or guinea bissau or guyana or british guiana or haiti or hispaniola or honduras or hungary or india or indonesia or timor or iran or iraq or isle of man or jamaica or jordan or kazakhstan or kazakh or kenya or "democratic people's republic of korea" or republic of korea or north korea or south korea or korea or kosovo or kyrgyzstan or kirghizia or

kirgizstan or kyrgyz republic or kirghiz or laos or lao pdr or "lao people's democratic republic" or latvia or lebanon or lebanese republic or lesotho or basutoland or liberia or libya or libyan arab jamahiriya or lithuania or macau or macao or republic of north macedonia or macedonia or madagascar or malagasy republic or malawi or nyasaland or malaysia or malay federation or malaya federation or maldives or indian ocean islands or indian ocean or mali or malta or micronesia or federated states of micronesia or kiribati or marshall islands or nauru or northern mariana islands or palau or tuvalu or mauritania or mauritius or mexico or moldova or moldovian or mongolia or montenegro or morocco or ifni or mozambique or portuguese east africa or myanmar or burma or namibia or nepal or netherlands antilles or nicaragua or niger or nigeria or oman or muscat or pakistan or panama or papua new guinea or new guinea or paraguay or peru or philippines or philipines or phillipines or phillippines or poland or "polish people's republic" or portugal or portuguese republic or puerto rico or romania or russia or russian federation or ussr or soviet union or union of soviet socialist republics or rwanda or ruanda or samoa or pacific islands or polynesia or samoan islands or navigator island or navigator islands or "sao tome and principe" or saudi arabia or senegal or serbia or seychelles or sierra leone or slovakia or slovak republic or slovenia or melanesia or solomon island or solomon islands or norfolk island or norfolk islands or somalia or south africa or south sudan or sri lanka or ceylon or "saint kitts and nevis" or "st. kitts and nevis" or saint lucia or "st. lucia" or "saint vincent and the grenadines" or saint vincent or "st. vincent" or grenadines or sudan or suriname or surinam or dutch guiana or netherlands guiana or syria or syrian arab republic or tajikistan or tadjikistan or tadzhikistan or tadzhik or tanzania or tanganyika or thailand or siam or timor leste or east timor or togo or togolese republic or tonga or "trinidad and tobago" or trinidad or tobago or tunisia or turkey or turkmenistan or turkmen or uganda or ukraine or uruguay or uzbekistan or uzbek or vanuatu or new hebrides or venezuela or vietnam or viet nam or middle east or west bank or gaza or palestine or yemen or yugoslavia or zambia or zimbabwe or northern rhodesia or global south or africa south of the sahara or sub-saharan africa or subsaharan africa or africa, central or central africa or africa, northern or north africa or northern africa or magreb or maghrib or sahara or africa, southern or southern africa or africa, eastern or east africa or eastern africa or africa, western or west africa or western africa or west indies or indian ocean islands or caribbean or central america or latin america or "south and central america" or south america or asia, central or central asia or asia, northern or north asia or northern asia or asia, southeastern or southeastern asia or south eastern asia or southeast asia or south east asia or asia, western or

western asia or europe, eastern or east europe or eastern europe or developing country or developing countries or developing nation? or developing population? or developing world or less developed countr* or less developed nation? or less developed population? or less developed world or lesser developed countr* or lesser developed nation? or lesser developed population? or lesser developed world or under developed countr* or under developed nation? or under developed population? or under developed world or underdeveloped countr* or underdeveloped nation? or underdeveloped population? or underdeveloped world or middle income countr* or middle income nation? or middle income population? or low income countr* or low income nation? or low income population? or lower income countr* or lower income nation? or lower income population? or underserved countr* or underserved nation? or underserved population? or underserved world or under served countr* or under served nation? or under served population? or under served world or deprived countr* or deprived nation? or deprived population? or deprived world or poor countr* or poor nation? or poor population? or poor world or poorer countr* or poorer nation? or poorer population? or poorer world or developing econom* or less developed econom* or lesser developed econom* or under developed econom* or underdeveloped econom* or middle income econom* or low income econom* or lower income econom* or low gdp or low gnp or low gross domestic or low gross national or lower gdp or lower gnp or lower gross domestic or lower gross national or lmic or lmics or third world or lami countr* or transitional countr* or emerging economies or emerging nation?).ti,ab,sh,kf.

259 257 not 258

260 (social prescri$ or refer$).mp. or "Social Determinants of Health"/

261 (program or treatment or management or education or support or physical exercise or aerobic or physical activity or leisure-time or exercise or sport or leisure activit* or physical fitness or training or physical performance or weight loss or weight reduction or BMI or body weight or body mass index or obesity or overweight or adiposity or smoking or tobacco or cigarette or social support or loneliness).mp.

262 260 and 261

263 259 and 262
